# Association between decreased grip strength in preschool children and the COVID-19 pandemic: an observational study from 2015 to 2021

**DOI:** 10.1186/s40101-023-00321-8

**Published:** 2023-03-24

**Authors:** Atsumu Yuki, Yumi Tamase, Mika Nakayama

**Affiliations:** 1 Faculty of Education, Kochi University, 2-5-1 Akebono, Kochi City, Kochi 780-8520 Japan; 2 Kuroshio Science Program, Graduate School of Integrated Arts and Sciences, Kochi University, 200 Monobe-Otsu, Nankoku City, Kochi 783-8502 Japan; 3 Kindergarten affiliated with the Faculty of Education, Kochi University, 10-26 Odu, Kochi City, Kochi 780-0915 Japan

**Keywords:** Muscle strength, Development of physical fitness, SARS-CoV-2

## Abstract

**Background:**

Coronavirus disease 2019 (COVID-19) has reduced people’s physical activity. It is essential to accumulate knowledge regarding the influence of COVID-19 on the stimulation of physical fitness and physical functions. Several studies have reported the effects of COVID-19 on physical fitness; however, there are very few reports regarding preschoolers. This study aimed to compare the physical fitness of preschoolers before and during the COVID-19 pandemic to clarify the effects of curtailment of outings implemented to control the pandemic on physical fitness among preschoolers.

**Methods:**

The subjects were 593 Japanese preschool children enrolled at a kindergarten during 2015–2019 and in 2021 who received a physical fitness test. Children enrolled in 2020 who did not receive a physical fitness test because of the COVID-19 pandemic were excluded. The physical fitness test included grip strength, standing long jump, and a 25-m run. The relationship between physical fitness level and survey year was analyzed using a general linear model, with grip strength and standing long jump as dependent variables, year of study as the independent variable, and sex and age in months as adjusted variables. Kruskal–Wallis test was used to analyze data for the 25-m run. Multiple comparisons were used to compare fitness levels between 2021 (during the COVID-19 pandemic) with levels in previous years.

**Results:**

Significant relationships were found between survey year and each of grip strength (*p* < 0.001), standing long jump (*p* < 0.05), and 25-m run (*p* < 0.001) among the overall subjects. Grip strength was significantly lower in 2021 compared with the 2016–2019 period. Similarly, sub-stratification analysis by sex showed that grip strength was lower in 2021 than in previous survey years, in both sexes. However, there was no difference in standing long jump or 25-m run times between before and during the pandemic among the overall subjects or according to sex.

**Conclusions:**

These findings indicate that the COVID-19 pandemic has had a negative effect on the development of muscle strength in preschoolers, and suggest the need to develop strategies that could promote the development of muscle strength of preschool children when limitations are placed on activity during prolonged infectious disease pandemics.

**Supplementary Information:**

The online version contains supplementary material available at 10.1186/s40101-023-00321-8.

## Background

Coronavirus disease (COVID-19) caused by severe acute respiratory syndrome-associated coronavirus-2 (SARS-CoV-2), of which the first outbreak was in 2019 in Wuhan, China, severely impacted people's wellbeing. Its impacts on physical fitness include a decrease in physical activity resulting from lockdowns and the refraining of the general population from non-essential outings [1], and an associated increase in obesity and decline in physical fitness [2, 3]. Impairments of physical function and fitness following SARS-CoV-2 infection have also been reported [4]. In view of the after-effects of COVID-19, it is essential to accumulate knowledge concerning the stimulation of physical fitness and physical functions.

Several reports have indicated a decline in physical fitness and physical function in school-aged children following the COVID-19 pandemic [5–9]. According to the National Survey on Physical Fitness, Exercise Ability and Exercise Habits in Japan in 2021 [10], the total physical fitness scores of both elementary and junior high school students were lower in 2021 compared to the results of the 2019 survey. Effects of existing risk factors such as decreased exercise time and increased screen time, as well as those of activity limitation due to COVID-19, have been suggested as reasons for the decline in physical fitness. Knowledge of the effects of COVID-19 behavioral restrictions on children’s fitness is accumulating; however, there are very few reports regarding preschoolers [11]. The aim of this study was to compare the physical fitness of preschool children before and during the COVID-19 pandemic to clarify the effects of curtailment of outings implemented to control the pandemic on physical fitness among preschoolers.

## Methods

### Subjects

The subjects were 708 preschool children enrolled in kindergartens affiliated with the Faculty of Education of the National University in Kochi city between 2015 and 2021. Of these, all children enrolled in 2020 (*n* = 93), who did not receive a physical fitness test due to the COVID-19 pandemic, and those with missing variables for the analysis (*n* = 22) were excluded from the study. The final number of subjects included in the analysis was 593. The number of participants in each year from 2015 to 2021 (excluding 2020) was 107, 103, 104, 106, 90, and 83 respectively; 301 boys and 292 girls. The median and interquartile range of age in months and distribution of subjects by grade and sex are shown in Table 1.

**Table 1:** Distribution of subjects by year of survey

		2015 (*n* = 107)	2016 (*n* = 103)	2017 (*n* = 104)	2018 (*n* = 106)	2019 (*n* = 90)	2021 (*n* = 83)	*χ*^2^	*p*
Age, months		63, 53–70	59, 53–71	62, 57–68	62, 53–71	61, 56–70	61, 51–68	1.11	0.953
Grade, *n*	First (age 3 years)	25	27	20	27	20	26		
	Second (age 4 years)	41	38	46	33	37	25	8.05	0.624
	Third (age 5 years)	41	38	38	46	33	32		
Sex, *n*	Boys	56	58	52	46	44	45	4.22	0.518
	Girls	51	45	52	60	46	38		

Age data are presented as the median and interquartile range

*p* values were obtained using Kruskal–Wallis test or *χ*-square test

### Physical fitness test

The physical fitness tests included grip strength, standing long jump, and a 25-m run. Grip strength was measured twice each, alternating left and right, using an infant grip dynamometer (T.K.K.5825; Takei Scientific Instruments, Niigata, Japan) and the average value was calculated from the maximum values for each of the left and right sides. The standing long jump was performed using a sheet with lines drawn at 1 cm intervals (KH-164, Kaneya Industry, Osaka, Japan). The distance of the jump was measured from the take-off line to the nearest point of contact during landing, and was performed twice, with the higher recorded distance used for analysis. The 25-m run was measured using a runway set at 30 m and the children were instructed to run through the 30 m line at the highest speed possible. The test was performed once and recorded to the nearest 0.1 s (SVAJ001; Seiko Watch Corporation, Tokyo, Japan).

### Statistical analysis

Statistical analysis was performed using EZR ver. 1.60 (Saitama Medical Center, Jichi Medical University, Saitama, Japan), which is a graphical user interface for R 4.2.1 (The R Foundation for Statistical Computing, Vienna, Austria), and SAS ver. 9.4 (SAS Institute, Inc., Cary, NC, USA). Normality of the data was tested using the Shapiro–Wilk test. Analysis of the relationship between physical fitness and survey year was performed using a general linear model with grip strength and standing long jump as dependent variables, year of survey as an independent variable, and sex and age in months as adjusted variables. Multiple comparisons were made using the Dunnett–Hsu method with the year 2021 as the control group. For analysis of the 25-m run data, which were not normally distributed, the Kruskal–Wallis test was used with the year of the survey as the independent variable. Multiple comparisons were compared between 2021 and earlier years using the Dwass–Steele–Critchlow–Fligner test. Partial η^2^ was calculated for the effect size of the main effect with general linear model and *r* for the effect size of multiple comparisons.

## Results

Table 2 shows the relationship between the results of the physical fitness test and survey year. In the overall subjects, there was a significant association between grip strength, standing long jump, 25-m run, and survey year. Multiple comparisons revealed that grip strength was lower in 2021 than in 2016–2019. However, there was no difference in standing long jump and 25-m run in 2021 compared to earlier years. The sex-stratified analysis showed a significant association between grip strength and 25-m run and survey year among the boys. Multiple comparisons revealed that grip strength was lower in 2021 than in 2016–2018. There was no difference in 25-m run in 2021 compared to earlier years. Among the girls, there was a significant association between grip strength, standing long jump, 25-m run, and survey year. Multiple comparisons revealed that grip strength was lower in 2021 than in 2015–2019. The standing long jump was lower in 2021 than in 2016.

**Table 2:** Relationship between physical fitness and the COVID-19 pandemic

	Year of survey								
	2015	2016	2017	2018	2019	2021	F or *χ*^2^	*p*	partial η^2^
Overall (*n* = 593)	*n* = 107	*n* = 103	*n* = 104	*n* = 106	*n* = 90	*n* = 83			
Grip strength, kg	7.7 ± 0.2 (0.13)	8.9 ± 0.2*** (0.40)	8.9 ± 0.2*** (0.41)	8.6 ± 0.2*** (0.34)	8.3 ± 0.2*** (0.29)	7.2 ± 0.2	12.14	< 0.001	0.09
Standing long jump, cm	90.8 ± 1.4 (0.04)	94.0 ± 1.5 (0.15)	89.5 ± 1.5 (< 0.00)	87.3 ± 1.5 (0.08)	92.9 ± 1.6 (0.11)	89.6 ± 1.6	2.72	0.019	0.02
25-m run, s	6.6, 5.9–7.4 (– 0.11)	6.8, 6.0–7.5 (– 0.03)	6.6, 6.1–7.3 (– 0.07)	6.6, 6.2–7.5 (– 0.04)	7.2, 6.7–8.1 (0.19)	6.6, 6.3–7.6	22.23	< 0.001	–
Boys (*n* = 301)	*n* = 56	*n* = 58	*n* = 52	*n* = 46	*n* = 44	*n* = 45			
Grip strength, kg	7.8 ± 0.3 (0.01)	9.1 ± 0.3** (0.32)	9.4 ± 0.3*** (0.36)	9.1 ± 0.3** (0.32)	8.6 ± 0.3 (0.20)	7.8 ± 0.3	6.15	< 0.001	0.09
Standing long jump, cm	94.5 ± 2.1 (0.01)	94.2 ± 2.1 (0.01)	95.4 ± 2.2 (0.03)	90.4 ± 2.3 (0.13)	96.8 ± 2.4 (0.08)	94.5 ± 2.3	0.85	0.517	0.01
25-m run, s	6.6, 5.8–7.4 (– 0.11)	6.8, 6.3–7.5 (0.04)	6.3, 5.9–6.9 (– 0.19)	6.6, 6.2–7.2 (– 0.04)	7.0, 6.3–7.9 (0.14)	6.6, 6.2–7.6	14.15	0.015	–
Girls (*n* = 292)	*n* = 51	*n* = 45	*n* = 52	*n* = 60	*n* = 46	*n* = 38			
Grip strength, kg	7.6 ± 0.3* (0.27)	8.6 ± 0.3*** (0.50)	8.4 ± 0.3*** (0.46)	8.0 ± 0.2*** (0.37)	8.0 ± 0.3*** (0.39)	6.5 ± 0.3	6.91	< 0.001	0.11
Standing long jump, cm	87.1 ± 2.0 (0.08)	94.9 ± 2.1** (0.34)	83.3 ± 1.9 (0.05)	84.3 ± 1.8 (0.02)	88.9 ± 2.1 (0.15)	84.8 ± 2.3	4.40	< 0.001	0.07
25-m run, s	6.5, 6.0–7.5 (– 0.11)	6.9, 6.0–7.5 (– 0.09)	6.9, 6.3–7.7 (0.03)	6.7, 6.1–7.5 (–0.06)	7.5, 6.7–8.1 (0.23)	6.8, 6.3–7.8	15.39	0.009	–

Values are mean ± standard error or median and interquartile range

Values in parentheses indicate effect size (*r*)

^*^p < 0.05; ^**^p < 0.01; ^***^p < 0.001 vs. survey in 2021

## Discussion

The present study investigated whether the COVID-19 pandemic caused a decline in physical fitness among preschool children in Japan by comparing physical fitness tests of preschoolers before and during the COVID-19 pandemic. The results showed that grip strength was lower during the COVID-19 pandemic than before the pandemic.

This study appears to be the first to report a reduction in grip strength in preschool children due to the COVID-19 pandemic. Several studies have evaluated the association between the COVID-19 pandemic and grip strength in children and adolescents. A study of adolescents reported an association between the COVID-19 pandemic and decreased grip strength in male students [3]. However, studies of school-aged children close to the age of the present subjects have reported that the COVID-19 pandemic and related lockdown were not associated with decreased grip strength [5, 8, 9]. The inconsistency between the present results and the previous results of school-aged children may be due to differences in the year in which the fitness tests were performed during the COVID-19 pandemic, rather than to differences in school grades between the studies. The World Health Organization (WHO) declared the COVID-19 outbreak as a global pandemic on 11 March 2020 [12]. These previous studies began to assess grip strength within one year at the latest after the pandemic declaration [5, 8, 9]. In contrast, the present study used fitness test data collected approximately 18 months after the declaration of the pandemic. Consistent with studies that reported a decrease in physical activity in preschool children that was associated with the COVID-19 pandemic [13–16], the present subjects might have been exposed for a relatively longer time to low levels of physical activity. Although the present study did not assess physical activity, some studies have reported that COVID-19 behavioral restriction has increased sedentary behavior in preschool children [15, 17]. Although there are no consistent results on whether sedentary behavior in preschool children inhibits the development of grip strength [18–20], if sedentary behavior caused by COVID-19 behavioral restrictions had increased in the present subjects, this could possibly be the reason for the inhibition of grip strength development. Furthermore, Japan experienced repeated waves of the COVID-19 pandemic during this 18-month period [21] during which restrictions on activities were ordered. In addition, attendance at kindergarten and leaving the home were prohibited for children infected with SARS-CoV-2 or identified as close contacts of a SARS-CoV-2-infected person. These environments might have been risk factors for increased sedentary behavior in preschool children.

Decreased grip strength during the COVID-19 pandemic might have been caused by insufficiency of physical development resulting from measures taken against COVID-19, including behavioral restrictions. However, as height and weight data were available only for third-grade children (aged 5 years), it was difficult to provide sufficient results. Therefore, to examine whether there was any association of stunted growth due to the COVID-19 pandemic with reduced grip strength, we performed additional analysis using the complete data for third-grade children to examine differences in body mass index between the survey years. The results showed no differences in body mass index between the survey years (Supplementary Table S1). This finding indicates that in the specific case of third-grade children, the decline in grip strength in 2021 was not due to impairment of physical development.

Although the trend of lower grip strength during the COVID-19 pandemic than before the pandemic was generally consistent for both sexes, the effect size was greater in girls than in boys. Therefore, the impact of the COVID-19 pandemic on grip strength might have been stronger in girls than in boys. No previous studies have assessed the effects of the COVID-19 pandemic on grip strength in preschool children. A study of US children aged 5–13 years reported that girls were exposed to longer sitting times than boys in the early post-pandemic period [22]. A study of Canadian children reported that fewer girls aged 5–11 years were achieving sufficient levels of physical activity after the pandemic than boys of the same age group [23]. These reports suggest the interesting possibility that undesirable lifestyle habits may have appeared in girls specifically. However, as studies assessing muscle strength and physical activity in preschool children are limited, further research is essential to clarify this issue.

The present study found no association of the COVID-19 pandemic with reduced jump and sprint performance. A previous study that compared fundamental motor skills such as standing long jump and 25-m run before and after the COVID-19 pandemic in 608 Japanese preschool children reported no change in the standing long jump before and after the pandemic, in all age groups. A decline in the 25-m running record after the COVID-19 pandemic was reported only in the 5-year-old group [11]. The present results appear to generally support the findings of this previous report [11]. It is well known that jumping and sprinting ability are affected by coordinated motions as well as by lower limb muscular strength. Coordination of these motions occurs through play in early childhood. In addition, moderate-to-vigorous physical activity (MVPA) is positively associated with the development of jumping and sprinting ability [18, 19]. A study of preschool children aged 3–5 years in 14 countries found no significant change in MVPA in preschool children between before and during the COVID-19 pandemic [17]. In addition, a study of Japanese preschool children reported no change in MVPA on weekends before and during the COVID-19 pandemic [15]. We did not assess physical activity in the present study; however, it is possible that COVID-19 behavioral restrictions might not have reduced MVPA in preschool children. Nevertheless, a minor discrepancy remains, as the results of the 25-m run in the 5-year-old population differ between studies [11]. Among the present female subjects, there were differences between the standing long jump in 2016 and in 2021. Furthermore, several studies of children aged 6 years and older have noted that standing long jump ability was reduced following the COVID-19 pandemic [7, 8]. Further research is necessary to address these conflicting findings.

This study has several limitations. First, the study design did not follow individuals. Therefore, the effect of the COVID-19 pandemic on the development of the physical fitness of preschool children is unknown. Second, as the study was based on one kindergarten in a rural city, the results cannot be generalized. Third, data on physical fitness for 2020 and on physical activity were not obtained. These data might be necessary to further elucidate the association between physical fitness of preschoolers and the COVID-19 pandemic. Furthermore, other background factors affecting physical fitness were not adequately considered in the analysis. Play with physical movement is considered particularly important for the physical development of preschool children. There might have been changes in play behavior, such as a shift from outdoor play to indoor play and fewer opportunities to play with friends.

## Conclusion

This study examined the impact of the COVID-19 pandemic on physical fitness of preschool children by comparing physical fitness data obtained in 2015–2019 with those in 2021. Grip strength was significantly lower in 2021 than in 2016–2019. These findings indicate that the COVID-19 pandemic may have had a negative effect on the development of muscle strength in preschool children, and suggest the need to develop strategies that could promote the development of muscle strength in preschool children when prolonged infectious disease pandemics occur.

## Supplementary Information

**Additional file 1: SupplementalTable S1.** Relationship between body mass index and the COVID-19pandemic in five-year-old children.

## Data Availability

The datasets generated during and/or analyzed during the current study are available from the corresponding author on reasonable request.

## Abbreviations

COVID-19: Coronavirus disease 2019
SARS-CoV-2: Severe acute respiratory syndrome-related coronavirus-2
