# Supplementary material for: Association between decreased grip strength in preschool children and the COVID-19 pandemic: an observational study from 2015 to 2021
Source: J Physiol Anthropol. 2023 Mar 24;42:4. doi: 10.1186/s40101-023-00321-8 (PMC10036968; doi:10.1186/s40101-023-00321-8)
Supplement: Supplementary file 1 — Additional file 1: SupplementalTable S1. Relationship between body mass index and the COVID-19pandemic in five-year-old children. [file 40101_2023_321_MOESM1_ESM.docx]

**Supplementary Table 1 Relationship between body mass index and the COVID-19 pandemic in five-year-old children**

|  |  | **Year of survey** | | | | | | | | |
| --- | --- | --- | --- | --- | --- | --- | --- | --- | --- | --- |
|  |  | **2015** | **2016** | **2017** | **2018** | **2019** |  | **2021** | **χ^2^** | **p** |
| Body mass index (kg/m^2^) | Overall (n=228) | 15.1, 14.5–15.9  (–0.25) | 15.1, 14.1–16.1  (–0.28) | 15.0, 14.5–16.1  (–0.20) | 15.2, 14.2–15.9  (–0.27) | 15.1, 14.5–16.4  (–0.18) |  | 15.7, 15.2–16.5 | 7.62 | 0.179 |
|  | Boys (n=116) | 15.5, 14.5–16.1  (–0.16) | 14.9, 14.4–16  (–0.28) | 14.9, 14.4–16.1  (–0.25) | 15.4, 14.8–16.3  (–0.16) | 15.7, 14.9–16.6  (–0.05) |  | 15.7, 15.3–16.1 | 5.93 | 0.313 |
|  | Girls (n=112) | 14.8, 14.5–15.6  (–0.30) | 15.1, 14–16.2  (–0.26) | 15.5, 14.5–16.4  (–0.12) | 14.6, 14–15.9  (–0.35) | 14.7, 14.3–15.6  (–0.29) |  | 15.8, 14.9–16.6 | 6.91 | 0.228 |

Values are median and interquartile range.

p-values were obtained using Kruskal–Wallis test.

Values in parentheses indicate effect size (r)
